# Angiotensin-converting enzyme insertion/deletion polymorphism association with obesity and some related disorders in Egyptian females: a case-control observational study

**DOI:** 10.1186/s12986-016-0127-5

**Published:** 2016-10-12

**Authors:** Tarek K. Motawi, Olfat G. Shaker, Nancy N. Shahin, Nancy M. Ahmed

**Affiliations:** 1 Department of Biochemistry, Faculty of Pharmacy, Cairo University, Cairo, Egypt; 2 Department of Medical Biochemistry and Molecular Biology, Faculty of Medicine, Cairo University, Cairo, Egypt; 3 Department of Biochemistry, Faculty of Pharmacy, Nahda University, Beni-Suef, Egypt

**Keywords:** ACE I/D polymorphism, Obesity, Hypertension, Insulin resistance, Metabolic syndrome, Egypt

## Abstract

**Background:**

According to the WHO report in 2015, obesity is the fifth leading cause of death worldwide, and the prevalence of Egyptian female obesity is 37.5 %. Since obesity is highly influenced by genetics, and adipose tissue renin-angiotensin system is over-activated in obesity, the effect of angiotensin-converting enzyme (ACE) insertion/deletion (I/D) polymorphism on obesity and related disorders was studied in several populations, because of its effect on ACE activity. Our objective was to study the association of ACE I/D polymorphism with obesity and certain related disorders, namely hypertension, insulin resistance and metabolic syndrome, in Egyptian females.

**Methods:**

Eighty female volunteers were recruited, blood pressure and body measurements were recorded and a fasting blood sample was obtained for the quantitation of glucose, lipid profile, insulin, leptin and identification of ACE I/D polymorphs. Subjects were grouped based on hypertension and obesity states. Comparisons of continuous parameters were made with independent sample *t*-test between two groups. The frequencies of ACE genotypes and alleles, and the association between gene polymorphism and metabolic parameters were assessed using chi-square or Fisher's exact test.

**Results:**

Genotype frequencies were in Hardy-Weinberg equilibrium for all groups. Genotype distribution did not differ significantly between controls and cases of all the studied disorders. Although DD carriers had apparently higher parameters of blood pressure, lipid profile and insulin resistance, only diastolic blood pressure was almost significant (*p* = 0.057). I-carriers were significantly less susceptible to hypertension than DD carriers having normal waist/hip ratio (*p* = 0.007, OR = 17.29, CI = 1.81–164.96) and normal conicity index (*p* = 0.024, OR = 7.00, CI = 1.36–35.93). In DD genotype carriers, a significant association was found between insulin resistance and high body mass index (*p* = 0.004, OR = 8.89, CI = 1.94–40.71), waist circumference (*p* = 0.003, OR = 9.63, CI = 2.14–43.36) and waist/height ratio (*p* = 0.034, OR = 6.86, CI = 1.25–37.61), although the variations in percentages between DD and I-carriers were not high enough to conclude an effect of ACE I/D on such an association.

**Conclusions:**

In this sample of Egyptian females, ACE I/D polymorphism was not significantly associated with obesity nor with any of its related disorders studied. The I allele seemed protective against hypertension in subjects with normal, not high, waist/hip ratio and conicity index compared to DD genotype carriers.

## Background

The worldwide prevalence of obesity more than doubled between 1980 and 2014. According to the WHO health profile of Egypt, the prevalence of adult female obesity in Egypt (37.5 %) was much higher than the Eastern Mediterranean regional average (26.4 %) which, in turn, was higher than the global average (20.5 %). On the other hand, male obesity showed less prevalence in Egypt (20.3 %), but comparable regional and global averages (27.5 % and 24 %, respectively). The profile also mentioned that the mean body mass index (BMI) in Egyptian adult females was 30.7 while the global average for females was 24.6 [1].

Raised BMI is a major risk factor for non-communicable diseases such as cardiovascular diseases, which were the leading cause of death in 2012, diabetes, musculoskeletal disorders and some cancers. Most of the world's population live in countries where overweight and obesity cause higher mortality than underweight [1]. Hypertension is the most frequent disease that accompanies obesity, and BMI is a significant predictor of high blood pressure even in non-obese populations. The converse is also true, a higher blood pressure predicts future obesity in Egyptian adolescents [2]. Obesity is also associated with glucose intolerance and fasting hyperinsulinemia [3]. In addition, abdominal obesity and hypertension were the most common abnormalities responsible for the high prevalence of metabolic syndrome (MetS) in women in many populations [4].

Leptin is a peptide expressed and secreted by adipocytes. It serves an “adipostat” function that regulates fat mass by decreasing food intake and increasing energy expenditure or thermogenesis. Leptin was found to be related to obesity, hypertension and insulin resistance [5].

Angiotensin-converting enzyme (ACE), a key enzyme in the renin-angiotensin system (RAS), catalyzes the conversion of the decapeptide angiotensin I to the potent vasopressor octapeptide angiotensin II (Ang II) [6–8]. The ACE gene consists of 26 exons and spans 21 kb on the long arm of chromosome 17 (17q23.3). It has a common “indel” polymorphism characterized by the insertion/presence (I allele) or deletion/absence (D allele) of a 287-bp Alu repeat sequence in intron 16. ACE activity in individuals with DD genotype is 65 % higher than those with II genotypes, while subjects with ID genotype have 31 % higher ACE activity than II homozygotes [6–8]. The components of over-activated RAS produced by adipocytes provide a potential pathway through which obesity leads to hypertension and insulin resistance [9].

Research on the genetic architecture of complex traits such as obesity should be designed and analyzed in a sex-specific way so that data can be separately examined in both men and women [10]. In most WHO regions, women had roughly double the obesity prevalence of men [1]. Another reason is sex-related physical dimorphism generally conceptualized as android versus gynoid distributions of adipose tissue; with females having an hourglass body shape (smaller waist/hip ratio "WHR") whilst males having an inverted triangle shape (larger WHR) [10].

ACE I/D polymorphism association with hypertension has been extensively studied among different populations, and associations with obesity and insulin resistance have also been studied to a lesser extent. In this context, ACE I/D polymorphism association studies in Egyptians are very limited, with few reports linking it to hypertension and diabetes [11, 12], but none of them addressed its correlation with obesity. Also, very few studies were conducted on Arabs and Africans, but not on Egyptians, that concurrently assessed all these disorders in relation to ACE I/D polymorphism [13, 14]. Moreover, there are no reports, to date, on the association of ACE gene I/D polymorphism with MetS in Arabs or Africans.

Therefore, the aim of the present work was to investigate the association of obesity and its related disorders, namely hypertension, insulin resistance and MetS, with ACE I/D polymorphism in Egyptian females comparing it with Arabic and African studies.

## Methods

The study was conducted on 80 adult non-pregnant females chosen from the outpatient clinic at the Internal Medicine Department, Kasr El-Aini Hospital, affiliated to Cairo University. Participants ranged in age from 30–65 years. Subjects who had hematologic, renal, hepatic or thyroid diseases were excluded. For each participant, full medical and family history, anthropometric measurements and blood pressure were recorded, in addition to taking a fasting blood sample (samples were collected during the period from April to December 2010).

### Anthropometric measurements

Body weight was measured with light clothing and shoes off using electronic scales. Non-stretchable tape was used to take height, which was measured in an upright standing position without shoes, waist circumference (WC), which was measured at the midpoint between the bottom of the ribs and the top of the iliac crest at the level of the umbilicus, and hip circumference which was measured at the largest posterior extension of the buttocks.

### Blood pressure

For systolic and diastolic blood pressure (SBP and DBP) measurements, auscultation method was performed on the left upper arm in sitting position after resting for at least 15 min using a mercury sphygmomanometer and a stethoscope, according to the American Heart Association recommendations [15].

### Blood sampling

A 12 h-fasting blood sample was then taken and divided into 3 portions for further analysis: 2 ml of blood was collected on EDTA for the detection of ACE gene polymorphism, 1 ml was fluorinated for the determination of fasting blood glucose level, and 2 ml was used to separate serum for the assay of insulin, leptin and lipid profile.

### Molecular biology techniques

Genomic DNA was extracted and purified from whole peripheral blood samples using QIAamp DNA mini kit (Qiagen Inc., Valencia, CA, USA) and stored at −80 °C in aliquots until required. Amplification of the ACE gene was carried out by polymerase chain reaction (PCR) using Taq PCR Master Mix (Qiagen, Valencia, CA, USA) and T-Gradient thermal cycler (Biometra, Germany). The sequence of primers used for amplification of ACE was 5'-CTGGAGACCACTCCCATCCTTTCT-3' (forward) and 5'-GATGTCGCCATCACATTCGTCAGAT-3' (reverse). Amplification of the I allele is sometimes suppressed in ID genotype samples giving rise to mistyping of ID as DD in approximately 5 % of cases. To avoid such mistyping, samples of the DD genotype were subjected to a second independent PCR with primers that recognize an insertion-specific sequence: 5'-TGGGACCACAGCGCCCGCCACTAC-3' (forward) and 5'-TCGCCAGCCCTCCCATGCCCATAA-3' (reverse). Electrophoresis was then performed on 2 % agarose gels stained with Red-Safe and the electrophoretic products were visualized using ultra-violet light trans-illumination. The PCR product was either 490 bp insertion (I allele) or 190 bp deletion (D allele). The second PCR with the insertion-specific primers yielded a 335 bp DNA product in the presence of the I allele, and no product for homozygous DD samples.

### Biochemical laboratory investigations

For the assay of plasma glucose level, the kit for enzymatic-colorimetric determination, supplied by STANBIO laboratory (Boerne, TX, USA), was used. Serum triglycerides (TG) level was determined by an enzymatic-colorimetric method using a kit supplied by PIONTE SCIENTIFIC Inc. (Canton, MI, USA). Serum total cholesterol (TC) level was determined by an enzymatic-colorimetric method using a kit supplied by LABKIT (Chemelex, SA, Spain). Serum high density lipoproteins (HDL) level was determined using HDL cholesterol precipitating kit supplied by CHRONOLAB (Barcelona, Spain). Low density lipoproteins (LDL) level was calculated using the Friedewald equation: LDL = TC – HDL – (TG/5).

### Enzyme-linked immunosorbent assay (ELISA)

Serum level of leptin was determined using a solid phase Enzyme Amplified Sensitivity Immunoassays (EASIA) kit supplied by DiaSource Europe S.A (Nivelles, Belgium). Its detection limit was 0.04 ng/ml, the inter-assay coefficient of variance (CV) was 10.2–12.7 % and the intra-assay CV was 3.5–13.3 %. Insulin was assayed using an ELISA kit supplied by Quantikine (Shanghai, China) that employed a quantitative sandwich enzyme immunoassay technique. Its detection limit was 0.881 pmol/L, the inter-assay CV was 6.9–7.5 % and the intra-assay CV was 3.7–4.0 %.

### Calculated indices

Several indices were calculated for each subject including indices for blood pressure [mean arterial pressure "MAP" and pulse pressure "PP" [16], insulin resistance [homeostasis model assessment "HOMA", quantitative insulin sensitivity check index "QUICKI" and fasting glucose insulin ratio "FGIR" [17], adiposity [body mass index “BMI”, waist/hip ratio “WHR”**,** waist/height ratio "WHtR", and conicity index "C-index, a measure of abdominal obesity calculated as: Waist circumference(m)/0.109 √ [weight(Kg)/ height(m)]" [5]] and lipid ratios [TC/HDL and LDL/HDL].

### Subjects grouping

The studied individuals were classified into four groups based on both BMI and hypertension state [obese hypertensive patients (OH, *n* = 28), obese normotensive patients (ON, *n* = 23), lean hypertensive patients (LH, *n* = 12) and lean normotensive controls (LN, *n* = 17)]. Subjects were also divided into 2 categories based on each of hypertension, BMI, WC, WHR, WHtR, HOMA and MetS. The cutoffs used for categorization were: SBP ≥ 140 or DBP ≥ 90 for hypertension [15], BMI ≥ 26 [18], WC ≥ 92 cm [19], WHR ≥ 0.85 [20], WHtR ≥ 0.52 [21], C-index ≥ 1.31 [22], and HOMA ≥ 3.5 [23]. Metabolic syndrome was considered for patients having at least 3 of 5 criteria: increased WC " ≥ 92 cm for the Egyptian population", SBP ≥ 130 mmHg and/or DBP ≥ 85 mmHg, fasting glucose ≥ 100 mg/dl, TG ≥ 150 mg/dl and HDL < 50 mg/dl in women [4].

### Statistical analyses

Prior to association analyses, the significance of deviations of observed genotype frequencies from those predicted by the Hardy–Weinberg equation were evaluated using chi square tests. Data are presented as mean (standard deviation) for quantitative variables, and as numbers (percentages) for categorical variables. The sample size achieved moderate effect size of 0.4 and the power of statistics was 0.8 at *p* < 0.05. Comparisons of the continuous parameters were made with independent sample *t*-test between two groups. The frequencies of ACE genotypes and alleles, and the association between the gene polymorphism and metabolic parameters were assessed using the chi-square test. Fisher's exact test was used instead of chi-square test for comparing categorical data when more than 20 % of the expected frequencies were less than 5. For such significant associations, risk estimate was presented as unadjusted odds ratios (OR) and confidence intervals (CI). A value of *p* < 0.05 (two-tailed) was considered significant. All the statistical analyses were carried out using SPSS (Chicago, IL, USA) software, version 17.0 for Microsoft Windows.

## Results

The studied sample of the Egyptian population comprised 80 females whose ages ranged from 30–65 years, with an average age of 48.8 years. There was no significant difference in age among different groups. The distribution of polymorphs within this 80-subject sample was 62.5 % DD (50 subjects), 31.25 % ID (25 subjects) and 6.25 % II (5 subjects). Allele frequencies were 78.1 % for the D allele and 21.9 % for the I allele. Genotype distributions within the pooled sample and among different groupings were all in Hardy-Weinberg equilibrium in cases as well as in controls (Table 1).

**Table 1:** Hardy-Weinberg equilibrium for ACE I/D genotypes

Groupings (n)	Observed frequencies			Expected frequencies			*χ* ^2^	*p*
	DD	ID	II	DD	ID	II		
All 80 subjects	50	25	5	48.8	27.3	3.8	0.590	0.443
OH (28)	19	7	2	18.1	8.8	1.1	1.212	0.271
ON (23)	14	7	2	13.3	8.4	1.3	0.616	0.433
LH (12)	9	3	0	9.2	2.6	0.2	0.245	0.621
LN (17)	8	8	1	8.5	7.1	1.5	0.302	0.583
Hypertensive (40)	28	10	2	27.2	11.6	1.2	0.720	0.396
Normotensive (40)	22	15	3	21.8	15.5	2.8	0.040	0.842
High BMI (51)	33	14	4	31.4	17.3	2.4	1.915	0.178
Normal BMI (29)	17	11	1	17.5	10.1	1.5	0.238	0.686
High WC (57)	37	16	4	35.5	18.9	2.5	1.379	0.240
Normal WC (23)	13	9	1	13.3	8.4	1.3	0.130	0.718
High WHR (55)	37	15	3	36.0	17.0	2.0	0.755	0.385
Normal WHR (25)	13	10	2	13.0	10.1	2.0	0.002	0.968
High WHtR (69)	43	21	5	41.5	24.0	3.5	1.101	0.294
Normal WHtR (11)	7	4	0	7.4	3.3	0.4	0.543	0.461
High C-index (44)	32	10	2	31.1	11.8	1.1	0.998	0.318
Normal C-index (36)	18	15	3	18.1	14.9	3.1	0.003	0.959
Insulin Resistant (62)	39	21	2	39.5	20.0	2.5	0.168	0.681
Insulin Sensitive (18)	11	4	3	9.4	7.2	1.4	3.583	0.058
MetS (45)	27	15	3	26.5	16.1	2.5	0.210	0.647
No MetS (35)	23	10	2	22.4	11.2	1.4	0.402	0.526

*OH* obese hypertensive, *ON* obese normotensive, *LH* lean hypertensive, *LN* lean normotensive, *BMI* body mass index, *WC* waist circumference, *WHR* waist/hip ratio, *WHtR* waist/height ratio, *C-index* conicity index, *MetS* metabolic syndrome, *DD* homozygous deletion genotype, *ID* heterozygous insertion deletion genotype, *II* homozygous insertion genotype, *χ* ^2^: chi-square test statistic

Despite apparently higher values of blood pressure and lipid profile parameters in DD genotype carriers, no significant difference was observed between DD genotype and I-carriers for all tested parameters, namely blood pressure, lipid profile, insulin resistance and weight-related parameters, using *t*-test for comparison, except for diastolic blood pressure which showed an almost significant elevation in DD genotype carriers as compared to I-carriers (*p* = 0.057, Table 2).

**Table 2:** Comparison of different parameters between DD and I-carriers using *t*-test

	DD (50)	ID + II (30)	*t*	*p*
Age (years)	49.62 (7.42)	46.57 (9.60)	1.50	0.14
SBP (mmHg)	133.20 (19.69)	128.17 (16.74)	1.17	0.25
DBP (mmHg)	85.30 (12.43)	81.00 (7.47)	1.93	0.057
MAP (mmHg)	101.27 (14.45)	96.72 (9.97)	1.66	0.10
PP (mmHg)	47.90 (10.26)	47.17 (11.87)	0.29	0.77
Weight (Kg)	76.26 (21.43)	77.35 (26.06)	−0.20	0.84
Height (cm)	154.72 (7.16)	156.03 (5.39)	−0.87	0.39
WC (cm)	101.26 (16.15)	98.97 (15.73)	0.62	0.53
Hip (cm)	110.84 (14.49)	111.27 (18.40)	−0.12	0.91
BMI (Kg/m^2^)	31.68 (8.37)	31.56 (9.88)	0.06	0.96
WHR	0.91 (0.08)	0.89 (0.10)	0.95	0.35
WHtR	0.65 (0.10)	0.63 (0.10)	0.88	0.38
C-index	1.34 (0.09)	1.31 (0.13)	0.97	0.33
Leptin (ng/ml)	9.50 (6.26)	9.60 (8.07)	−0.06	0.95
TC (mg/dl)	166.00 (30.50)	159.07 (30.86)	0.98	0.33
TG (mg/dl)	87.90 (35.36)	76.80 (24.55)	1.51	0.13
LDL (mg/dl)	111.64 (27.78)	105.70 (30.29)	0.89	0.37
HDL (mg/dl)	37.14 (9.50)	37.87 (7.58)	−0.36	0.72
TC/HDL	4.69 (1.22)	4.36 (1.18)	1.16	0.25
LDL/HDL	3.19 (1.10)	2.93 (1.07)	1.02	0.31
Glucose (mg/dl)	103.88 (20.90)	104.40 (18.47)	−0.11	0.91
Insulin (μIU/ml)	18.81 (4.72)	17.59 (4.93)	1.10	0.27
HOMA	4.87 (1.82)	4.56 (1.69)	0.77	0.44
QUICKI	0.306 (0.01)	0.308 (0.01)	−0.81	0.42
FGIR	5.81 (1.77)	6.30 (1.89)	−1.17	0.25

Data are presented as mean (SD)

*SBP* systolic blood pressure, *DBP* diastolic blood pressure, *MAP* mean arterial pressure, *PP* pulse pressure, *WC* waist circumference, *BMI* body mass index, *WHR* waist/hip ratio, *WHtR* waist/height ratio, *C-index* conicity index, *TC* total cholesterol, *TG* triglycerides, *LDL* low density lipoproteins, *HDL* high density lipoproteins, *HOMA* homeostasis model of assessment, *QUICKI* quantitative insulin sensitivity check index, *FGIR* fasting glucose insulin ratio, *DD* homozygous deletion genotype, *ID* heterozygous insertion deletion genotype, II: homozygous insertion genotype, t: *t*-test statistic

In order to apply the chi-square test of association, we converted the continuous data of blood pressure, certain obesity indices, HOMA and MetS into categorical data so that subjects could be distributed into two groups for each parameter. The results of the association tests are summarized in Table 3 where the significance level exceeded 0.05 for all tested groups based on blood pressure, BMI, WC, WHR, WHtR, C-index, HOMA and MetS. Groups based on being obese, hypertensive or both had apparently higher frequencies of the DD genotype than controls but such differences did not reach statistical significance.

**Table 3:** Association of ACE(I/D) genotype/allele frequencies with hypertension, obesity, insulin resistance and metabolic syndrome

Groups (n)	DD	ID + II	Significance	D allele	I allele	Significance
OH (28)	19 (67.9 %)	9 (32.1 %)	*χ* ^2^ = 2.898 *p* = 0.41	45 (80.4 %)	11 (19.6 %)	*χ* ^2^ = 2.64 *p* = 0.45
ON (23)	14 (60.9 %)	9 (39.1 %)		35 (76.1 %)	11 (23.9 %)	
LH (12)	9 (75.0 %)	3 (25.0 %)		21 (87.5 %)	3 (12.5 %)	
LN (17)	8 (47.1 %)	9 (53.9 %)		24 (70.6 %)	10 (29.4 %)	
Obese BMI (51)	33 (64.7 %)	18 (35.3 %)	*χ* ^2^ = 0.29 *p* = 0.59	80 (78.4 %)	22 (21.6 %)	*χ* ^2^ = 0.015 *p* = 0.90
Normal BMI (29)	17 (58.6 %)	12 (41.4 %)		45 (77.6 %)	13 (22.4 %)	
Large WC (57)	37 (64.9 %)	20 (35.1 %)	*χ* ^2^ = 0.49 *p* = 0.48	90 (78.9 %)	24 (21.1 %)	*χ* ^2^ = 0.16 *p* = 0.69
Normal WC (23)	13 (56.5 %)	10 (43.5 %)		35 (76.1 %)	11 (23.9 %)	
Large WHR (55)	37 (67.3 %)	18 (32.7 %)	*χ* ^2^ = 1.71 *p* = 0.19	89 (80.9 %)	21 (19.1 %)	*χ* ^2^ = 1.6 *p* = 0.21
Normal WHR (25)	13 (52.0 %)	12 (48.0 %)		36 (72.0 %)	14 (28.0 %)	
Large WHtR (69)	43 (62.3 %)	26 (37.7 %)	Fisher's Exact: *p* = 1.00	107 (77.5 %)	31 (22.5 %)	*χ* ^2^ = 0.20 *p* = 0.65
Normal WHtR (11)	7 (63.6 %)	4 (36.4 %)		18 (81.8 %)	4 (18.2 %)	
Large C-index (44)	32 (72.7 %)	12 (27.3 %)	*χ* ^2^ = 3.26 *p* = 0.071	74 (84.1 %)	14 (15.9 %)	*χ* ^2^ = 3.07 * p* = 0.08
Normal C-index (36)	18 (50.0 %)	18 (50.0 %)		51 (70.8 %)	21 (29.2 %)	
Hypertensive (40)	28 (70.0 %)	12 (30.0 %)	*χ* ^2^ = 1.92 *p* = 0.17	66 (82.5 %)	14 (17.5 %)	*χ* ^2^ = 1.79 *p* = 0.18
Normotensive (40)	22 (55.0 %)	18 (45.0 %)		59 (73.8 %)	21 (26.2 %)	
Insulin resistant (62)	39 (62.9 %)	23 (37.1 %)	*χ* ^2^ = 0.02 *p* = 0.89	99 (79.8 %)	25 (20.2 %)	*χ* ^2^ = 0.95 *p* = 0.33
Insulin sensitive (18)	11 (61.1 %)	7 (38.9 %)		26 (72.2 %)	10 (27.8 %)	
Positive MetS (45)	27 (60.0 %)	18 (40.0 %)	*χ* ^2^ = 0.27 *p* = 0.6	69 (76.7 %)	21 (23.3 %)	*χ* ^2^ = 0.26 *p* = 0.61
No MetS (35)	23 (65.7 %)	12 (34.3 %)		56 (80.0 %)	14 (20.0 %)	

Data are presented as number (percentage), n (%)

*OH* obese hypertensive subjects, *ON* obese normotensive subjects, *LH* lean hypertensive subjects, *LN* lean normotensive control subjects, *BMI* body mass index, *WC* waist circumference, *WHR* waist/hip ratio, *WHtR* waist/height ratio, *C-index* conicity index, *MetS* metabolic syndrome, *DD* homozygous deletion genotype, *ID* heterozygous insertion deletion genotype, *II* homozygous insertion genotype, *D* deletion allele, *I* insertion allele

Studying the possible relationship between obesity and hypertension, we found that among the different obesity indices studied, only WHR and C-index showed significant difference between hypertensive and normotensive subjects (*p* < 0.05, Table 4). Also, patients having higher WHR and WC had significantly higher blood pressure parameters than those with normal WHR or WC (both at *p* < 0.05), whereas other obesity classifications did not show that difference except for C-index that showed significant correlation with pulse pressure only (Table 5). As shown in Table 6, we also found that this high risk of being hypertensive in patients with high WHR was comparable in both DD and I-carriers (62.16 % and 61.11 % hypertensives, respectively). On the other hand, it was noticed that normal WHR subjects had much less odds of developing hypertension in I-carriers (only 8.3 % hypertensives) than in DD subjects (38.46 % hypertensives). Such effect of the I-allele in lowering the susceptibility of normal WHR subjects to hypertension was highly significant at *p* = 0.007 with odds ratio (OR) = 17.29 and confidence interval (CI) = 1.813–164.96. The same protective effect of the I-allele against hypertension was noticed when using the C-index to represent the obesity state, with comparable percentages of hypertensives in larger C-index DD and I-carriers (59.38 % and 66.67 %, respectively), while chances of being hypertensive were significantly lower in normal C-index I-carriers (22.22 %) than the corresponding DD counterparts (50.0 %) at *p* = 0.024 with OR = 7.00 and CI = 1.36–35.93. When using other indices to represent the obesity state, there was no significant *χ* ^2^ association between obesity and hypertension at any polymorph comparison (Table 6).

**Table 4:** Comparison of certain obesity-related parameters between groups based on hypertension and insulin resistance

	Hypertensive (40)	Normotensive (40)	Insulin resistant (62)	Insulin sensitive (18)
Weight (Kg)	76.36 (22.65)	76.98 (23.86)	80.1 (21.29)	64.86 (25.81)
	*t* = −0.12, *p* = 0.91		*t* = 2.55, *p* = 0.013*	
WC (cm)	102.28 (14.53)	98.53 (17.21)	102.63 (15.09)	92.72 (16.8)
	*t* = 1.05, *p* = 0.30		*t* = 2.39, *p* = 0.019*	
Hip (cm)	110.23 (15.92)	111.78 (16.16)	113.53 (15.14)	102.28 (16.01)
	*t* = −0.43, *p* = 0.67		*t* = 2.74, *p* = 0.008*	
BMI (Kg/m^2^)	31.55 (8.18)	31.72 (9.67)	32.96 (8.42)	27.07 (9.23)
	*t* = −0.09, *p* = 0.93		*t* = 2.56, *p* = 0.013*	
WHR	0.93 (0.09)	0.88 (0.07)	0.905 (0.085)	0.908 (0.089)
	*t* = 2.83, *p* = 0.01*		*t* = −0.11, *p* = 0.91	
WHtR	0.66 (0.09)	0.63 (0.11)	0.66 (0.1)	0.6 (0.1)
	*t* = 1.21, *p* = 0.23		*t* = 2.17, *p* = 0.033*	
C-index	1.36 (0.10)	1.30 (0.10)	1.324 (0.105)	1.336 (0.104)
	*t* = 2.43, *p* = 0.02*		*t* = −0.38, *p* = 0.708	

Data are presented as mean (SD)

*: significant difference at *p* < 0.05

*WC* waist circumference, *BMI* body mass index, *WHR* waist/hip ratio, *WHtR* waist/height ratio, *C-index* conicity index, t: *t*-test statistic

**Table 5:** Comparison of leptin and blood pressure parameters between different obesity classifications, using *t*-test

	Leptin (μIU/ml)	SBP (mmHg)	DBP (mmHg)	MAP (mmHg)	PP (mmHg)
Large BMI	12.43 (6.96)	132.98 (16.43)	85.29 (10.36)	101.19 (11.85)	47.69 (9.73)
Normal BMI	4.17 (1.83)	128.21 (22.29)	80.71 (11.68)	96.55 (14.84)	47.50 (12.80)
	*t* = 8.06, *p* < 0.001*	*t* = 0.99, *p* = 0.33	*t* = 1.80, *p* = 0.08	*t* = 1.53, *p* = 0.13	*t* = 0.07, *p* = 0.95
Large WC	11.67 (7.10)	134.21 (17.37)	85.79 (10.51)	101.93 (12.25)	48.42 (10.40)
Normal WC	4.26 (1.97)	124.13 (20.26)	78.48 (10.60)	93.70 (13.45)	45.56 (11.80)
	*t* = 7.22, *p* < 0.001*	*t* = 2.24, *p* = 0.03*	*t* = 2.81, *p* = 0.01*	*t* = 2.65, *p* = 0.01*	*t* = 1.04, *p* = 0.30
Large WHR	9.45 (6.19)	134.55 (18.35)	85.18 (11.04)	101.64 (12.87)	49.38 (11.20)
Normal WHR	9.76 (8.59)	123.75 (17.59)	80.21 (10.27)	94.72 (12.49)	43.54 (8.78)
	*t* = −0.16, *p* = 0.78	*t* = 2.44, *p* = 0.02*	*t* = 1.88, *p* = 0.06	*t* = 2.22, *p* = 0.03*	*t* = 2.27, *p* = 0.03*
Large WHtR	10.51 (6.99)	132.54 (18.30)	84.42 (10.87)	100.46 (12.84)	48.12 (10.71)
Normal WHtR	3.47 (0.91)	123.64 (20.14)	79.09 (11.14)	93.94 (13.73)	44.55 (11.50)
	*t* = 7.95, *p* < 0.001*	*t* = 1.48, *p* = 0.14	*t* = 1.51, *p* = 0.14	*t* = 1.55, *p* = 0.13	*t* = 1.02, *p* = 0.31
Large C-index	9.07 (6.37)	133.75 (18.40)	83.64 (10.64)	100.34 (12.65)	50.11 (11.28)
Normal C-index	10.11 (7.63)	128.33 (18.86)	83.75 (11.55)	98.61 (13.69)	44.58 (9.52)
	*t* = −0.67, *p* = 0.51	*t* = 1.29, *p* = 0.20	*t* = −0.05, *p* = 0.96	*t* = 0.59, *p* = 0.56	*t* = 2.34, *p* = 0.02*

Data are presented as mean (SD)

*: significant difference at *p* < 0.05

*SBP* systolic blood pressure, *DBP* diastolic blood pressure, *MAP* mean arterial pressure, *PP* pulse pressure, *BMI* body mass index, *WC* waist circumference, *WHR* waist/hip ratio, *WHtR* waist/height ratio, *C-index* conicity index, t: *t*-test statistic

**Table 6:** Associations of obesity (based on different obesity indices) with hypertension and insulin resistance layered by I/D genotypes

Polymorph	Obesity	Hypertension		Insulin resistance	
		Hypertensive	Normotensive	Resistant	Sensitive
DD	Large WHR (37)	23 (62.2 %)	14 (37.8 %)	30 (81.1 %)	7 (18.9 %)
	Normal WHR (13)	5 (38.5 %)	8 (61.5 %)	9 (69.2 %)	4 (30.8 %)
		*χ* ^2^ = 2.19, *p* = 0.14		Fisher's exact: *p* = 0.45	
I-carriers	Large WHR (18)	11 (61.1 %)	7 (38.9 %)	14 (77.8 %)	4 (22.2 %)
	Normal WHR (12)	1 (8.3 %)	11 (91.7 %)	9 (75.0 %)	3 (25.0 %)
		Fisher's exact: *p* = 0.007* OR = 17.29, CI = 1.81–164.96		Fisher's exact: *p* = 1.00	
DD	Large C-index (32)	19 (59.4 %)	13 (40.6 %)	25 (78.1 %)	7 (21.9 %)
	Normal C-index (18)	9 (50.0 %)	9 (50.0 %)	14 (77.8 %)	4 (22.2 %)
		*χ* ^2^ = 0.41, *p* = 0.52		Fisher's exact: *p* = 1.00	
I-carriers	Large C-index (12)	8 (66.7 %)	4 (33.3 %)	9 (75.0 %)	3 (25.0 %)
	Normal C-index (18)	4 (22.2 %)	14 (77.8 %)	14 (77.8 %)	4 (22.2 %)
		Fisher's exact: *p* = 0.024* OR = 7.00, CI = 1.36–35.93		Fisher's exact: *p* = 1.00	
DD	Obese BMI (33)	19 (57.6 %)	14 (42.4 %)	31 (91.2 %)	3 (8.8 %)
	Normal BMI (17)	9 (52.9 %)	8 (47.1 %)	8 (50.0 %)	8 (50.0 %)
		*χ* ^2^ = 0.10, *p* = 0.75		Fisher's exact: *p* = 0.004* OR = 8.89, CI = 1.94–40.71	
I-carriers	Obese BMI (18)	9 (50.0 %)	9 (50.0 %)	15 (83.3 %)	3 (16.7 %)
	Normal BMI (12)	3 (25.0 %)	9 (75.0 %)	8 (66.7 %)	4 (33.3 %)
		Fisher's exact: *p* = 0.26		Fisher's exact: *p* = 0.39	
DD	Large WC (37)	23 (62.2 %)	14 (40.0 %)	33 (89.2 %)	4 (10.8 %)
	Normal WC (13)	5 (38.5 %)	8 (53.3 %)	6 (46.2 %)	7 (53.8 %)
		*χ* ^2^ = 2.19, *p* = 0.14		Fisher's exact: *p* = 0.003* OR = 9.63, CI = 2.14–43.36	
I-carriers	Large WC (20)	10 (50.0 %)	10 (50.0 %)	17 (85.0 %)	3 (15.0 %)
	Normal WC (10)	2 (20.0 %)	8 (80.0 %)	6 (60.0 %)	5 (40.0 %)
		Fisher's exact: *p* = 0.24		Fisher's exact: *p* = 0.18	
DD	Large WHtR (43)	25 (58.1 %)	18 (41.9 %)	36 (83.7 %)	7 (16.3 %)
	Normal WHtR (7)	3 (42.9 %)	4 (57.1 %)	3 (42.9 %)	4 (57.1 %)
		Fisher's exact: *p* = 0.68		Fisher's exact: *p* = 0.034* OR = 6.86, CI = 1.25–37.61	
I-carriers	Large WHtR (26)	12 (46.2 %)	14 (53.8 %)	19 (73.1 %)	7 (26.9 %)
	Normal WHtR (4)	0 (0 %)	4 (100 %)	4 (100 %)	0 (0 %)
		Fisher's exact: *p* = 0.13		Fisher's exact: *p* = 0.55	

Data are presented as number (percentage)

*: significant difference at *p* < 0.05

*DD* homozygous deletion genotype, *I-carriers* homozygous and heterozygous insertion genotypes, *BMI*: body mass index, *WC* waist circumference, *WHtR* waist/height ratio, *WHR* waist/hip ratio, *C-index* conicity index, *χ* ^*2*^ chi square, *OR* odds ratio, *CI* confidence intervals

Table 4 shows the significant difference in weight-related parameters between insulin-resistant and insulin-sensitive groups (*p*˂0.05), except for WHR and C-index. In addition, based on different obesity indices, we found that obesity classified by BMI, WC and WHtR showed highly significant *χ* ^2^ association with insulin resistance (using HOMA cut-off) only in the DD polymorph at *p* = 0.004, OR = 8.89 and CI = 1.94–40.71 for BMI, at *p* = 0.003, OR = 9.63 and CI = 2.14–43.36 for WC and at *p* = 0.034, OR = 6.86 and CI = 1.25–37.61 for WHtR. WHR and C-index showed no significant *χ* ^2^ association with insulin resistance (Table 6).

## Discussion

In the current study on Egyptian females, the association of ACE I/D polymorphism with obesity and several related disorders was studied. The ages of the studied subjects were generally homogenous. The distribution of polymorphs within this 80-subject sample was comparable to that reported in a previous study conducted on the Egyptian population [24].

As depicted in Tables 2 and 3, weight-related parameters and leptin did not differ significantly among ACE polymorphs (DD, I-carriers). Also, comparing the frequency of ACE genotypes or alleles between obese and lean groups did not show significant difference. So, no significant association between ACE I/D polymorphism and obesity was suggested. However, there seems to be some tendency towards obesity in DD genotype carriers as manifested by apparently higher DD genotype frequencies in obese subjects compared to their corresponding controls based on all obesity classifications. Also, there was no significant difference in blood pressure parameters between ACE polymorphs, although DD carriers had non-significantly higher blood pressure parameters than I-carriers with the difference in DBP being almost significant. Moreover, the distribution of the DD genotype was apparently higher in hypertensives than in normotensives but did not reach statistical significance. Thus, association of ACE I/D polymorphism with hypertension in the currently studied subjects cannot be suggested strongly enough. No significant difference was observed in glucose level or insulin resistance indices among ACE polymorphs, and the genotype and allele frequencies did not vary between insulin resistant and normal subjects. Lipid profile parameters did not differ significantly among genotypes, although subjects with the DD genotype showed slightly higher values than I-carriers. Finally, no association was found between ACE I/D polymorphism and MetS. Thus, it can be concluded that ACE I/D polymorphism was not significantly associated with obesity or any of the studied related disorders in this sample of Egyptian females.

Angiotensin-converting enzyme is a key player in the RAS as it catalyzes the conversion of angiotensin I to angiotensin II (Ang II). The ACE gene I/D polymorphism markedly affects ACE activity, where DD genotype carriers have 65 % higher ACE activity than those with the II genotype, and subjects with the ID genotype have 31 % higher activity than II homozygotes [6]. Owing to the principal role of ACE in the RAS, alterations in its activity, associated with its I/D polymorphism, will significantly affect the RAS. Over-activated systemic and adipose tissue RAS components provide a potential pathway through which obesity may lead to hypertension and insulin resistance, and through which ACE I/D polymorphism may be related to these disorders [9]. Activation of RAS may lead to obesity through the secretion of Ang II by mature adipocytes. Angiotensin II stimulates the differentiation of pre-adipocytes into mature adipocytes (hyperplasia) and increases lipid content of mature adipocytes (hypertrophy) by stimulation of lipogenesis and inhibition of lipolysis [25], and possibly through reducing adipose tissue blood flow [9]. Activation of the RAS may also contribute to insulin resistance in Ang II-responsive tissues [9], firstly by decreasing the percentage of small insulin-sensitive adipocytes in comparison to larger mature and relatively insulin-resistant cells, and secondly by reducing blood flow which reduces insulin and glucose delivery to the insulin-sensitive tissues [26]. Accordingly, the apparent predisposition of DD genotype carriers, in the present study, to obesity, hypertension and disrupted lipid profile could be justified by the aforesaid fact that the DD genotype has the highest ACE activity among all ACE I/D polymorphs. However, these relationships were only apparent and could not reach the accepted level of significance most likely due to our relatively small sample size.

Previous studies on ACE I/D polymorphism association with diseases mainly obesity and hypertension are too many worldwide. We herein compare our results with studies conducted on Arab and African populations which are remarkably limited in this regard. However, generally, the genetic association studies are sometimes controversial even within the same population [27, 28].

Very few studies considered the association of obesity with ACE I/D polymorphism in Arabs and Africans, where no association was found, similar to our findings, in Saudis [27] and Tunisians [13], while another study detected an association with the DD genotype in Saudis [28].

Regarding association studies of ACE I/D polymorphism with hypertension, only two studies were conducted on Egyptians and they associated the D allele with hypertension [11], mainly on noise exposure [12]. In Arabs, no correlation with hypertension was found in Emirates [29], in Lebanon [30] nor in Algeria [31]. When an association was found, hypertension was accompanied by other factors such as age in Lebanese [32], obesity in Tunisians [13] and Emiratis [14] or diabetes and obesity in Saudi patients [8].

Limited studies were also conducted in Arabs and Africans investigating the association of ACE I/D polymorphism with insulin resistance whereby no association was observed in Emiratis [14], Lebanese [33] nor in Tunisians [34]. On the other hand, the D allele was associated with insulin resistance in an earlier study on Egyptians [11]. Also, the DD genotype distribution was found to be significantly higher in diabetic patients in Tunis [6, 35] and in Bahrain [36]. The ACE I allele was found to be associated with diabetes among Kuwaitis [37].

As for association with lipid disorders, a Saudi study revealed that ACE gene I/D polymorphism has no role in predicting the occurrence and diagnosis of familial hypercholesterolemia [7]. Apart from that, no other studies were reported to investigate the association of ACE gene I/D polymorphism with lipid disorders nor with MetS in Arabs or Africans.

Studying the interaction between obesity and hypertension, we found that WHR and C-index were the best obesity indices to reflect hypertension, as they were significantly higher in hypertensive patients than in normotensive subjects while other obesity indices did not show such significance (Table 4). However, cases having high WC showed significantly higher blood pressure values than those with normal WC (Table 5). We also checked whether this interaction was affected by ACE I/D polymorphism. As previously discussed, although non-significant, the DD genotype was more prevalent than I-carriers in all cases than in controls, whether they were obese (ON), hypertensive (LH) or both (OH) (Table 3). Also, obese patients based on having high WHR or C-index had higher risk of being hypertensive than normal subjects, but to a similar extent in both DD and I-carriers. On the other hand, carrying the I-allele seemed more significantly protective against hypertension in subjects with normal WHR and C-index compared to their corresponding DD genotype counterparts (Table 6). It was previously reported that WHR and C-index show great accuracy for the detection of the risk of cardiovascular disease [38]. Fat stored in visceral depots (indicated by higher WHR) makes obese individuals more prone to complications than subcutaneous fat even at the same BMI. On the contrary, larger gluteo-femoral fat stores (indicated by smaller WHR) are protective [10].

We similarly checked the effect of ACE I/D on the interaction between obesity and insulin resistance. In DD genotype carriers, a significant association was found between insulin resistance and obesity as presented by BMI, WC and WHtR, although the variations in percentages between DD and I-carriers were not high enough to conclude a clear effect of ACE I/D on such an association (Table 6). Similarly, a study on Emiratis found no effect of obesity status on the association of ACE genotypes with diabetes [14]. Also, WC and BMI were previously reported to predict type 2 diabetes [3]. On the other hand, it was previously reported that the ACE D allele could raise diabetic risk in obese but not lean men [39].

A major limitation of this study is the relatively small sample size. Therefore, extending the investigation of the studied associations on a larger sample set is warranted to confirm that the present findings would replicate in other groups.

## Conclusions

Angiotensin-converting enzyme insertion/deletion polymorphism was not significantly associated with obesity in this sample of Egyptian females or with any of the studied disorders related to obesity, namely hypertension, insulin resistance and metabolic syndrome, regardless of the apparently higher DD genotype frequencies in obese and/or hypertensive patients compared to their normal counterparts. However, DD genotype carriers showed higher values of blood pressure parameters mainly diastolic blood pressure that was almost significant. In addition, the I allele significantly decreased the risk of hypertension in subjects with normal, not high, WHR and C-index in comparison to the DD genotype. On the other hand, a clear effect of ACE I/D polymorphism on the association of obesity and insulin resistance could not be established.

This is the first study to address the association of ACE I/D polymorphism with obesity and related disorders, specifically, hypertension, insulin resistance and metabolic syndrome, in Egyptian females. Very few studies addressed the association of this polymorphism with these disorders together in Arabs and Africans, but they all studied and analyzed both sexes together, while our study was sex-specific studying only females.

Further confirmatory studies on a larger sample number could be beneficial to support or contradict our results. We also suggest the inclusion of both males and females, but to be analyzed separately, to check whether ACE I/D polymorphism could justify the vast difference in obesity prevalence observed between Egyptian males and females.

## Abbreviations

ACE: angiotensin-converting enzyme
Ang II: angiotensin II
BMI: body mass index
CI: Confidence interval
C-index: conicity index
CV: coefficient of variance
D: deletion allele
DBP: diastolic blood pressure
DD: homozygous deletion genotype
EASIA: enzyme amplified sensitivity immunoassays
FGIR: fasting glucose insulin ratio
HDL: high density lipoproteins
HOMA: homeostasis model assessment
I: Insertion allele
I/D: insertion/deletion polymorphism
ID: heterozygous insertion deletion genotype
II: Homozygous insertion genotype
LDL: low density lipoproteins
LH: lean hypertensive patients
LN: lean normotensive controls
MAP: mean arterial pressure
MetS: metabolic syndrome
OH: obese hypertensive patients
ON: obese normotensive patients
OR: Odds ratio
PCR: polymerase chain reaction
PP: pulse pressure
QUICKI: quantitative insulin sensitivity check index
RAS: renin-angiotensin system
SBP: systolic blood pressure
TC: total cholesterol
TG: triglycerides
WC: waist circumference
WHO: World health organization
WHR: waist/hip ratio
WHtR: waist/height ratio
